# p53-dependent R-loop formation and HPV pathogenesis

**DOI:** 10.1073/pnas.2305907120

**Published:** 2023-08-23

**Authors:** Conor Winslow Templeton, Laimonis A. Laimins

**Affiliations:** ^a^Department of Microbiology-Immunology, Northwestern University Feinberg School of Medicine, Chicago, IL 60611

**Keywords:** HPV, p53, R-loops, life cycle

## Abstract

R-loops are trimeric RNA: DNA hybrids that are important physiological regulators of transcription; however, their aberrant formation or turnover leads to genomic instability and DNA breaks. High-risk human papillomaviruses (HPV) are the causative agents of genital as well as oropharyngeal cancers and exhibit enhanced amounts of DNA breaks. The levels of R-loops were found to be increased up to 50-fold in cells that maintain high-risk HPV genomes and were readily detected in squamous cell cervical carcinomas in vivo but not in normal cells. The high levels of R-loops in HPV-positive cells were present on both viral and cellular sites together with RNase H1, an enzyme that controls their resolution. Depletion of RNase H1 in HPV-positive cells further increased R-loop levels, resulting in impaired viral transcription and replication along with reduced expression of the DNA repair genes such as FANCD2 and ATR, both of which are necessary for viral functions. Overexpression of RNase H1 decreased total R-loop levels, resulting in a reduction of DNA breaks by over 50%. Furthermore, increased RNase H1 expression blocked viral transcription and replication while enhancing the expression of factors in the innate immune regulatory pathway. This suggests that maintaining elevated R-loop levels is important for the HPV life cycle. The E6 viral oncoprotein was found to be responsible for inducing high levels of R-loops by inhibiting p53’s transcriptional activity. Our studies indicate that high R-loop levels are critical for HPV pathogenesis and that this depends on suppressing the p53 pathway.

## Significance

High-risk human papillomaviruses (HPV) are the etiological agents of genital and oropharyngeal cancers. Although prophylactic vaccines are effective in blocking initial infection by these viruses, they are not effective against existing lesions. Understanding the mechanisms regulating HPV pathogenesis is therefore important for the identification of biomarkers and for the development of therapeutics. Our work demonstrates that high levels of trimeric RNA: DNA structures called R-loops are present in HPV-positive cells derived from low-grade cervical lesions as well as in squamous cell carcinomas. These elevated R-loop levels are necessary for both viral gene expression and DNA replication. Our studies demonstrate that R-loops are critical cellular regulators of HPV pathogenesis, and they may be useful as biomarkers for viral infection or therapeutic targets.

R-loops are trimeric structures consisting of an RNA-DNA hybrid and a displaced DNA strand that are formed during transcription (1–3). These structures form at promoters and sites of termination to regulate transcription (4–6); however, aberrant R-loop formation or turnover can lead to genomic instability and DNA breaks (7, 8). R-loop homeostasis is maintained by enzymes such as RNase H1 and H2 as well as senataxin (9). RNase H1 can degrade the RNA moiety in R-loops and controls the formation of aberrant R-loops (10, 11). Similarly, senataxin resolves R-loops that form at the 3′ end of the transcribed genes (12, 13). In normal cells, R-loops provide critical functions in regulating transcription, DNA damage repair, and other functions, while in cancers, they can result in genomic instability.

Human papillomaviruses (HPV) are the causative agents of cervical and most oropharyngeal cancers (14–17). HPVs infect cells in the basal layer of stratified epithelia and establish their genomes as nuclear episomes at about 100 copies per cell (18, 19). Initial studies indicated that the levels of R-loops were increased in HPV-positive cells but whether this had an effect on viral pathogenesis was unclear (20). In precancerous lesions, HPV genomes are maintained at a constant copy number in basal cells and replicated simultaneously with cellular DNA (21). As HPV-positive cells migrate from the basal layer they reenter S/G2 in suprabasal layers, where productive replication occurs in a process called amplification (22). Both stable maintenance replication and amplification depend on activation of the Ataxia-telangiectasia mutated (ATM) and Ataxia telangiectasia and Rad3 related (ATR) DNA repair pathways by the E6 and E7 viral proteins through induction of high levels of DNA breaks (23, 24). The preferential and rapid repair of these breaks in HPV DNAs is necessary for viral replication (20). DNA breaks result from the improper formation or resolution of R-loops but whether they contribute to HPV pathogenesis cells is unknown (25).

In this study, high levels of R-loops were detected on both viral and cellular sequences in cells that stably maintain episomes as well as squamous cell cervical carcinomas. The levels of R-loop regulatory enzymes such as RNase H1 were similarly increased. Knockdown of RNase H1 increased the levels of R-loops and at the same time impaired viral transcription and stable replication of HPV episomes. The resultant increased levels of R-loops also repressed expression of cellular genes involved in DNA damage repair including FANCD2 and ATR both of which are critical for viral replication (26, 27). The increased levels of R-loops were the result of E6 directed inhibition of p53 function. These studies identify R-loops as critical regulators of HPV pathogenesis, whose altered homeostasis is dependent upon repression of p53.

## Results

**Fig. 1.**
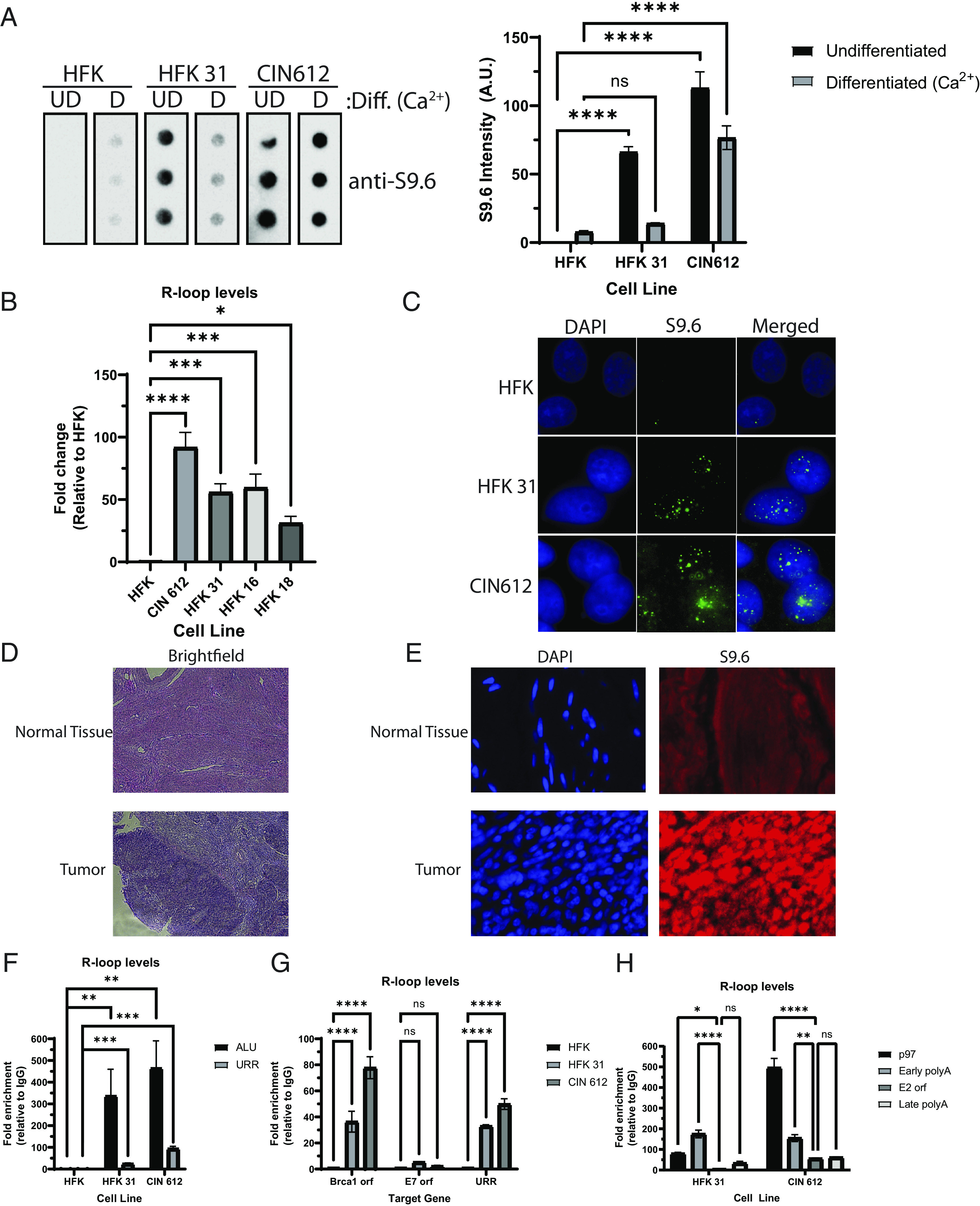
R-loops are enriched within nuclear puncta of HPV-positive keratinocytes. R-loop levels are increased in HPV-positive cells. (*A*) Dot-blot analysis of nucleic acid extracts from undifferentiated (UD) HFKs, HFK 31, and CIN 612 cells or differentiated (D) for 72 h in a high-calcium medium probed with S9.6 antibody showing increased levels in HPV-positive cells. *Left* panel shows dot-blot assays using technical triplicates. *Right* panel shows quantitation of data from 4 experiments, and data are plotted as the average of the mean with the error bars representing the SEM (*A*, *Right*; ns, not significant; ****P < 0.0001). (*B*) Graph showing quantitation of dot-blot assays of nucleic acids isolated from UD HFKs, CIN 612, HFK 31, HFK 16, and HFK 18 cells probed with the S9.6 antibody. Quantification of three biological replicates is plotted as the average and SEM (*B*; *P < 0.05; ***P < 0.001; ****P < 0.0001). (*C*) R-loops form as puncta within the nuclei of HPV-positive cells. Immunofluorescence analysis of UD HFKs, HFK 31, and CIN 612 cells using the S9.6 antibody (n = 3, a representative field is shown). (*D*) Hematoxylin and eosin staining was performed on paraffin-embedded tissue from high-grade cervical carcinomas that included normal tissue at adjacent margins. Cross-sections of the same tissue were used for immunofluorescence analysis with antibodies recognizing R-loops (S9.6) and DAPI (*E*). (*F*) DNA:RNA immunoprecipitation assays (DRIP) of HFKs, HFK 31, and CIN 612 cells were performed, and immunoprecipitated chromatin was quantified by qPCR analysis. R-loops form on the viral genome and ALU sequences in HPV-positive cells. Fold enrichment for each primer set over IgG is shown: (S9.6_x_/IgG_x_)/(S9.6_HFK_/IgG_HFK_), where x is Ct values from either HFK 31 or CIN 612. The error bars represent the SEM of six biological replicates. (*G*) DRIP assays for R-loops were performed and analyzed by qPCR for BRCA1 ORF, E7 ORF, or URR sequences in HFKs, HFK 31, or CIN 612 cells. Three biological replicates were analyzed (ns, not significant; *P < 0.05; **P < 0.01; ***P < 0.001; ****P < 0.0001). (*H*) DRIP assays using HFK 31, and CIN 612 cells were performed, and immunoprecipitated nucleic acid signals were quantified by qPCR analysis using primers from the p97 promoter, E2, early poly-A, and late poly-A regions of HPV 31. Fold enrichment for each primer set was measured as (S9.6_x_/IgG_x_).

To investigate what role, if any, R-loops might play in the HPV life cycle, the levels were examined in cells that stably maintain high-risk HPV 31 episomes. CIN612 cells were derived from a low-grade CIN biopsy while HFK-31 cells were generated by transfection of cloned viral sequences into primary human keratinocytes (HFK) (28, 29). DNA-RNA dot-blot analysis was performed utilizing an antibody that preferentially recognizes R-loops (S9.6) (30, 31), and 50 to 100 fold higher levels were detected in HPV-positive cells as compared to HFKs (Fig. 1*A*). In order to confirm that the increase was due to R-loops, samples were treated with RNase H to remove the RNA component prior to spotting on a membrane, and the signal was shown to be specific (*SI Appendix*, Fig. S1). To determine if the presence of high R-loop levels extended to other high-risk types, we performed S9.6 dot-blot assays using extracts from cells that stably maintain HPV 16 or 18 episomes and detected levels similar to those seen in HPV 31-positive cells (Fig. 1*B*). This indicates that high levels of R-loops are present in cells from multiple high-risk HPV types.

The life cycle of HPV is linked to the differentiation of the host keratinocyte (32), so it was important to determine if levels of R-loops in HFKs, HFK 31, and CIN 612 cells changed upon calcium-induced differentiation (Fig. 1*A*). The switch from low-to-high calcium media with keratinocytes grown in monolayer cultures induces differentiation that initiates around 48 h and peaks at 72 h (29). In HPV-positive cells, R-loop levels were reduced upon differentiation, and this was most pronounced in HFK-31 cells as compared to CIN 612. Interestingly, the levels of R-loops in HFKs were modestly increased upon differentiation in contrast to the decrease seen in HPV-positive cells. In this study, we focus our analyses on R-loop effects in undifferentiated (UD) cells. We next investigated where R-loops were localized in HPV-positive cells through immunofluorescence assays using the S9.6 antibody. R-loops were detected in multiple nuclear foci in UD HPV-positive cells, while only a small number of such foci were detected in HFKs (Fig. 1*C*). High levels of R-loops were also detected in tissue sections from biopsies of squamous cell cervical carcinomas and were absent in adjacent normal tissues (Fig. 1 *D* and *E*). Specificity of the S9.6 antibody in detecting R-loops from cross-linked tissues was addressed by treating tissues with RNase T and RNase III or RNase H and examining the staining of S9.6. In these tissues, S9.6 staining is sensitive to RNase H treatment and minimally to RNase T and III treatment (*SI Appendix*, Fig. S2).

**Fig. 2.**
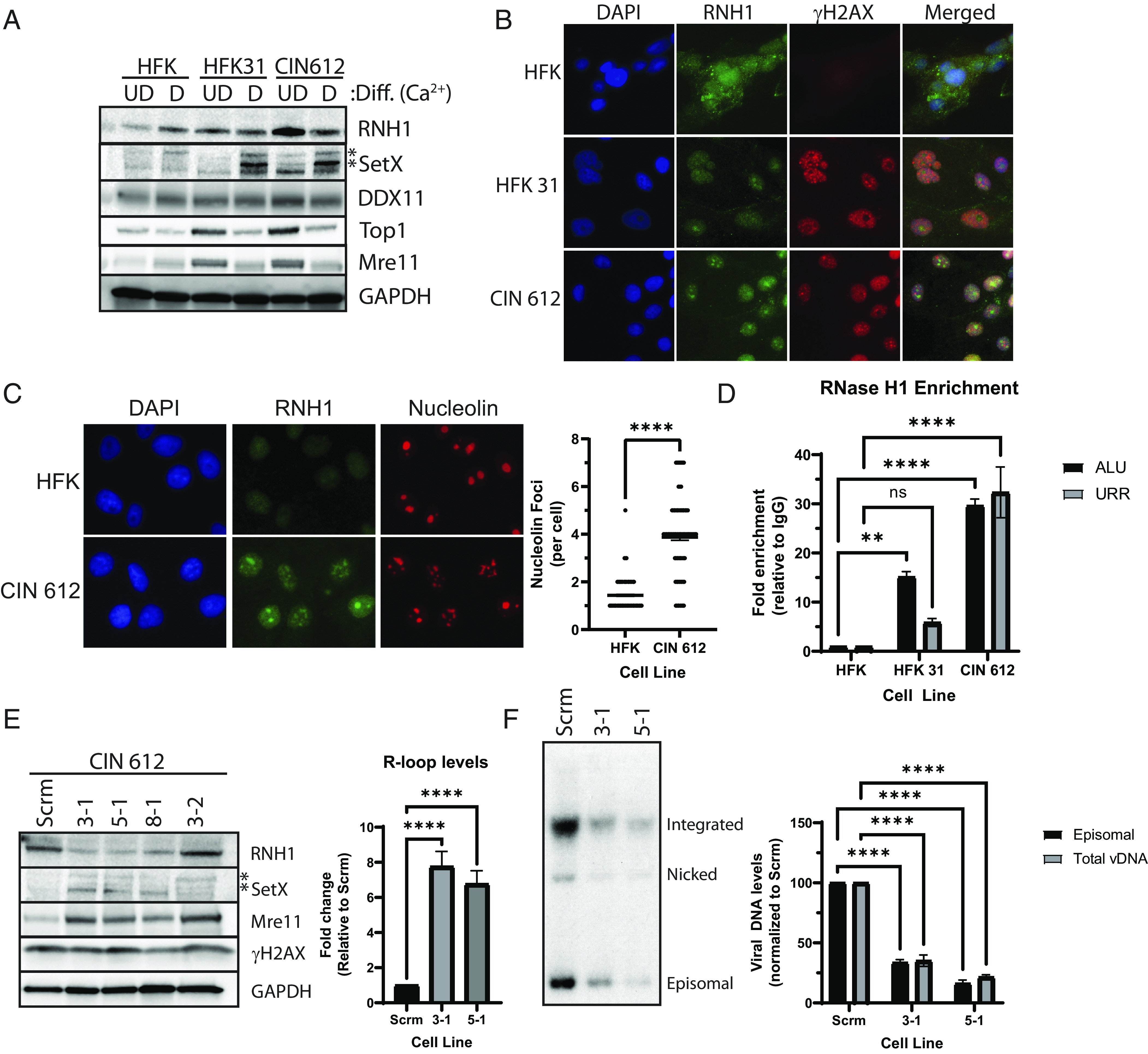
RNase H1 levels and localization are altered in HPV-positive cells. Levels of R-loop resolving enzymes are increased in UD HPV-positive cells (*A*). Western blot analysis for RNase H1 for HFK, HFK 31, and CIN 612 cells either UD or differentiated (D) for 72 h in a high-calcium medium (n = 3, a representative image is shown). Senataxin is posttranslationally modified and its modified forms are marked by asterisks (37, 38). Nonspecific bands are seen in other studies (39)). (*B* and *C*) RNase H1 is recruited to nuclear foci and nucleoli in HPV-positive cells. Immunofluorescence analysis of RNase H1 and gH2AX or RNase H1 and nucleolin in UD HFK, HFK 31, and CIN 612 cells (n = 3; a representative field is shown). Quantification of nucleolin foci in HFKs or CIN 612 cells is shown (*C*, *Right*) (n = 3, 150 cells) (*D*) RNase H1 is bound to both viral and cellular sequences in HPV-positive cells. Graph showing chromatin immunoprecipitation analysis of RNase H1 binding to ALU or HPV 31 URR sequences using extracts from HFKs, HFK 31, and CIN 612 cells. Fold enrichment is quantified as (RNase H1_x_/IgG_x_)/(RNase H1_HFK_/IgG_HFK_) where x are Ct values from either HFK 31 or CIN 612 cells (n = 3; ns, not significant; **P < 0.01; ****P < 0.0001). (*E*) Depletion of RNase H1 increases R-loop levels and impairs the maintenance of HPV genomes in UD CIN 612 cells. Cells were infected with retroviruses expressing either scramble control or 3 different shRNAs against RNase H1. *Left* panel shows western blot analysis of CIN 612 cells transduced with shRNA sequences targeting RNase H1 together with SETX, Mre11, gH2AX or GAPDH loading control. The *Right* panel shows S9.6 dot blots performed, showing that depletion of RNase H1 resulted in increased global R-loop levels. The data are plotted as the average of three biological replicates and the error bars are the SEM (****P < 0.0001). (*F*) RNase H1 depletion reduces HPV episomes within CIN 612 cells. Southern blot analysis showing cells stably expressing a scrambled control or shRNAs to RNase H1 depleted. A representative Southern blot is shown (*Left*), and the panel on the *Right* shows the genome copy numbers quantified as the average and SEM of three biological replicates (*Right*; ****P < 0.0001).

It was next important to determine if R-loops were associated with viral or cellular DNAs through DNA-RNA immunoprecipitation (DRIP) assays. This method uses the S9.6 antibody to precipitate R-loop complexes followed by qPCR for the DNA region of interest to measure binding (33). The formation of R-loops on the upstream regulatory region (URR) of the HPV genome was examined by DRIP analysis and compared to that seen on cellular sequences using ALU sequences as examples. High levels of R-loops were detected at both the URR of HPV 31 as well as at ALU sequences in both HFK-31 and CIN 612 cells (Fig. 1*F*). In contrast, very low levels of R-loops were observed at ALU sequences in HFKs. Since ALU sequences are present at high copy numbers within the cell, the presence of R-loops was also examined at promoter sequences around the BRCA1 gene, which has previously been reported to maintain R-loops and whose expression is up-regulated in HPV-positive cancers (34, 35). R-loops were detected within both coding and promoter regions of BRCA1 in the HFKs but were significantly increased in HPV-positive cells (Fig. 1*G*). These observations indicate that the enhanced levels of R-loops detected in HPV-positive cells were not the result of high-level formation only on viral episomes but were increased on cellular sequences as well. We next investigated if R-loops formed uniformly on the HPV genome or whether they were preferentially localized to the URR. For this analysis, R-loop formation within the coding sequences of E7 was compared to its association with the URR and found substantially higher levels present on the latter (Fig. 2*H*). DRIP analysis was also performed on the late and early polyA sites (36), the E2 orf together with the p97 promoter in the URR (Fig. 2*F*). Both the early polyA site and the p97 promoter region had significantly more R-loops compared to the E2 orf or the late polyA site. These data indicate that R-loops preferentially form at regions on the viral genome important for transcription of early viral genes.

### UD HPV-Positive Cells Have Increased Levels of Proteins Responsible for R-loop Resolution.

Since the formation and turnover of R-loops is regulated by enzymes such as RNase H1, senataxin, Mre11, DDX11, as well as TOP1 (40), it was important to determine whether the high levels of R-loops in HPV-positive cells were due to a reduction in the levels of these factors. Western blot analysis of UD HPV-positive cells demonstrated increased levels of all these factors compared to HFKs and paralleled the high levels of R-loops detected in these cells (Fig. 2*A*). Interestingly upon calcium-induced differentiation, the levels of RNase H1, Mre11, DDX11, and Top1 l decreased in the HPV-positive cells to the levels seen in HFKs even though the number of R-loops remained high. Only the levels of senataxin were found to remain elevated upon differentiation. This indicates that increased levels of R-loops in HPV-positive cells are not due to decreased steady-state levels of proteins responsible for the resolution of these structures.

### RNase H1 Is Enriched within the Nucleoli of HPV-Positive Cells.

While HPV-positive cells maintain a high level of RNase H1, it was possible that its subcellular localization was altered to inhibit its action. Immunofluorescence analysis of RNase H1 in HFKs demonstrated a pannuclear distribution with some cytoplasmic localization (Fig. 2 *B*, *Top* row). In CIN612 and HFK-31 cells, RNase H1 also exhibited a pannuclear distribution along with a number of densely staining foci. In addition, the total relative intensity of the signal increased compared to HFKs (Fig. 2*B*, rows 2 to 3). Since these RNase H1-positive foci resembled nucleoli, immunofluorescence for nucleolin, a marker of nucleoli (41), was performed and identified these areas as nucleoli enriched with RNase H1 (Fig. 2 *C*, *Left*). In addition, approximately 3 times more nucleolin puncta were observed in HPV-positive cells than HFKs (Fig. 2 *C*, *Right*). RNase H1 has been reported to act with RNA pol1 in mediating rRNA transcription and may explain this localization to nucleoli (42). In contrast the R-loop regulatory enzyme senataxin exhibited the same subcellular localization in both HFKs and HPV-positive cells with no recruitment to nucleoli though again total levels were increased (*SI Appendix*, Fig. S3). R-loops can also be associated with the formation of DNA breaks, but only modest colocalization with γH2AX, a surrogate marker for breaks (43), was observed (Fig. 2*B*). To determine whether RNase H1 was recruited to viral or cellular genomes, chromatin immunoprecipitation assays were performed. HFK 31 and CIN 612 cells exhibited high levels of RNase H1 on both the HPV URR and ALU sequences with the latter being increased in comparison to the HFKs (Fig. 2*D*). Interestingly, the amount of RNaseH1 binding per viral genome was significantly higher than the binding per ALU element. ALU elements are present at over 500 thousand copies per cell, while the HPV genomes are maintained at 50 to 100 copies.

### Depletion of RNase H1 Impairs HPV Genome Maintenance.

The presence of high levels of both RNase H1 and R-loops on viral genomes suggested they may play a role in the HPV life cycle, therefore the effect of depleting RNase H1 on viral replication and transcription was investigated. RNase H1 was stably depleted in CIN 612 cells by transduction with lentiviruses expressing shRNAs and western blot analysis showed levels were reduced by threefold relative to the scrambled shRNA control (Fig. 2*E*). This moderate reduction in RNase H1 levels, however, increased the amounts of R-loop levels by 5 to 10-fold, confirming its functions in the resolution of these structures (Fig. 2*E*). Since RNase H1 provides essential functions (44), the depleted cells could only be passaged up to 5 times before cell growth was arrested or suppression of RNase H1 expression was lost (10, 45). We, therefore, screened for effects on HPV replication by Southern blot analysis at the second passage after depletion and found HPV genomes were reduced by approximately fourfold (Fig. 2*F*). This indicates that stable replication of HPV episomes is impaired as a result of RNase H1 knockdown with the corresponding increase in R-loops.

**Fig. 3.**
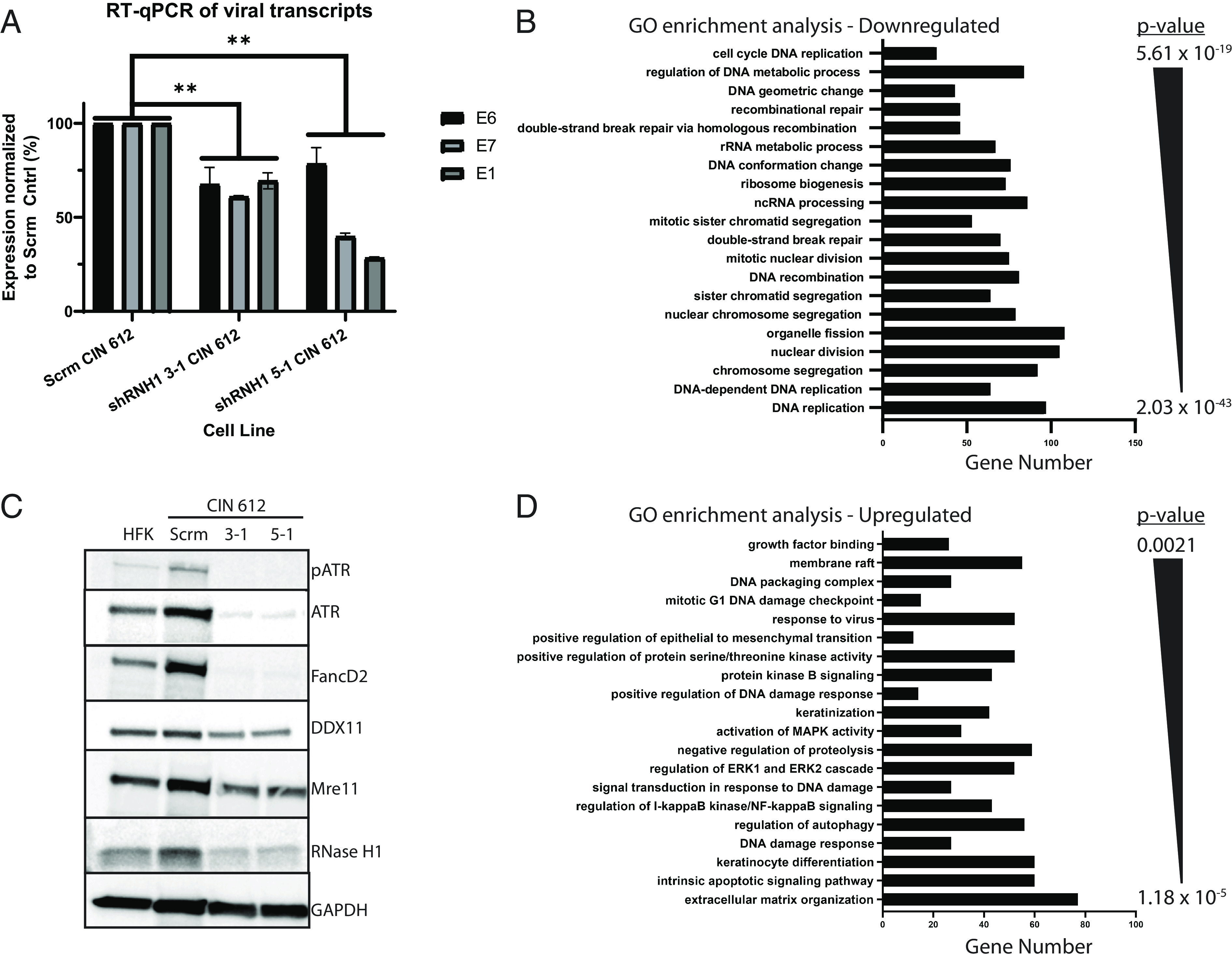
Depletion of RNase H1 alters viral and cellular gene expression in HPV-positive cells. (*A*) RT-qPCR was performed for viral transcripts encoding either E6, E7, or E1 from CIN 612 cells stably expressed scramble control shRNA (Scrm) or CIN 612 cells stably expressed shRNAs to RNase H1 (clone 3-1 or 5-1). The data are plotted as the average of three biological replicates and the error bars are the SEM (**P < 0.01). (*B*) RNA-seq analysis identifying major pathways whose expression is altered relative in cells with shRNAs to RNase H1 relative to scramble control CIN 612 cells. The graph represents the results of gene ontology enrichment analysis of the RNA-seq data (46). The number of genes in each pathway that exhibit decreased transcript levels comparing scrm control to shRNase H1 CIN 612 cells and *P*-value are plotted on the *y* axis at the right (*P* < 2.03 × 10^−43^ to *P* < 5.61 × 10^−19^). mRNA-sequencing analysis was performed in biological duplicate on each cell line: scrm CIN 612, shRNH1 3-1 CIN 612, and shRNH1 5-1 CIN 612. (*C*) Western blot analysis of representative genes down-regulated at the transcript level through RNA-seq. Levels of FANCD2, Mre11, RNase H1, and ATR levels decreased upon depletion of RNase H1 consistent with the RNA-seq data (n = 4, a representative image is shown). (*D*) Graph identifying pathways where expression of genes was increased in shRNase H1 CIN 612 cells relative to scramble control as determined by gene ontology enrichment analysis of RNA sequencing data (n = 4). *P*-values are shown on the *y* axis to the right (*P* < 1.18 × 10^−5^ to *P* < 0.0021).

R-loops can positively regulate both initiation and termination of transcription and can decrease levels if improperly formed or resolved. To determine what effect increased levels of R-loops had on viral transcription, Real time - (RT) qPCR was used to examine levels of E6, E7, and E1 transcripts in UD CIN 612 cells that were depleted of RNase H1 and compared to the scramble control. Depletion of RNase H1 decreased transcript levels by 30 to 50% suggesting that viral gene expression correlated with R-loop homeostasis (Fig. 3*A*).

Depletion of RNase H1, which leads to an increase in R-loops, could also affect cellular gene expression. To investigate how depletion of RNase H1 affected cellular gene expression, RNA-sequencing analysis (RNA-seq) was performed on CIN 612 cells that were depleted of RNase H1. Depletion of RNase H1 affected the expression of a number of cellular pathways including those involved in DNA replication, DNA damage response, and DNA recombination all of which impact the HPV life cycle (Fig. 3*B*). Of particular interest were reductions in factors such as FANCD2, ATR, and Mre11, which have been shown to be important for HPV replication (26, 27, 47). Western blot analysis confirmed that the reduced transcript levels of FANCD2, ATR, Mre11, and RNase H1 corresponded to lower protein steady-state levels (Fig. 3*C*). One important factor is FANCD2 as its knockdown impairs HPV replication in UD cells and may act together with ATR and Mre11 to explain the effects on viral replication upon depletion of RNase H1 (26). Other genes were up-regulated by RNase H1 depletion including those in the p53 arm of the DNA damage response (48), such as GADD45A, MDM2, and p21 (Fig. 3*D*). In addition, the expression of transcriptional repressors such as SNAIL along with members of the epithelial cell integrity pathway, including KLK5, KLK6, and KLK13 were similarly increased.

### Overexpression of RNase H1 Impairs HPV Genome Maintenance.

While knockdown of RNase H1 increased the levels of R-loops, overexpression can reduce levels (49). To investigate how increasing RNase H1 levels impacted viral functions, HPV-positive cells were transfected with a vector expressing a green fluorescent protein (GFP)-tagged RNase H1 that lacked the N-terminal mitochondrial localization signal, so it only localized to the nucleus (50). Increased levels of RNase H1 were confirmed by western blot analyses and localization to the nucleus was detected by immunofluorescence analyses. In addition, a decrease in the level of R-loops was confirmed by S9.6 dot-blot assays (*SI Appendix*, Fig. S4).

**Fig. 4.**
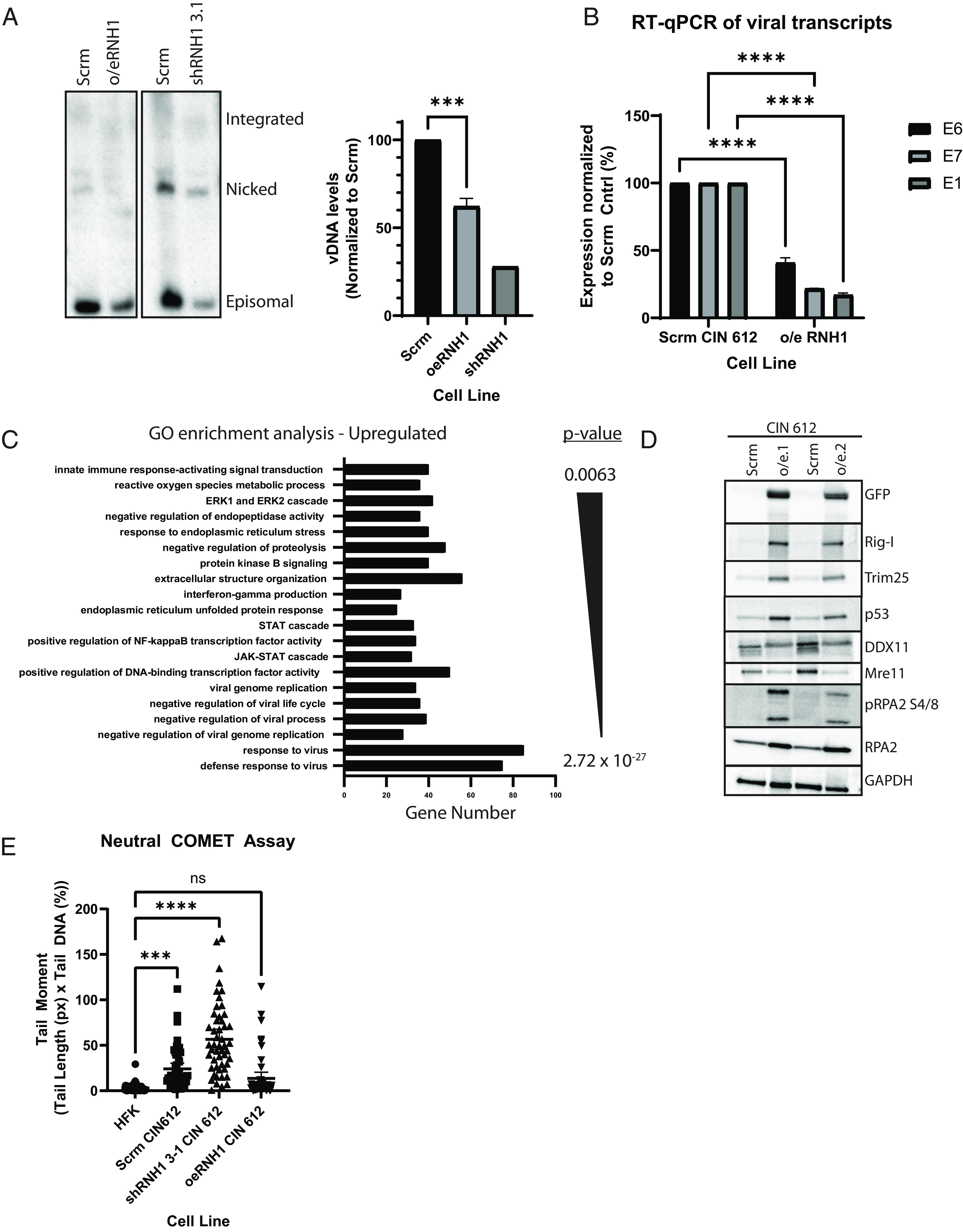
RNase H1 overexpression impairs genome maintenance and promotes the expression of immune response genes. (*A*) Overexpression of RNase H1 reduced HPV episomes. A Southern blot is shown of scrm CIN 612 cells compared to CIN 612 cells either overexpressing RNase H1 (o/e RNH1) or depleted of RNase H1. The associated graph shows data from the average of four biological replicates, and the error bars are the SEM (***P < 0.001). (*B*) Viral transcription is hindered by overexpression of RNase H1. RT-qPCR analysis of scrm or o/eRNH1 CIN 612 cells for viral transcripts encoding E1, E6, and E7 (n = 3; ****P < 0.0001). (*C*) RNA-seq analysis of cells overexpressing RNase H1 (o/e CIN612) showing pathways whose expression was increased as identified by gene ontology enrichment analysis (46). *P*-values are shown on the *y* axis to the right (*P* < 2.72 × 10^−27^ to *P* < 0.0063). (*D*) Western blot analysis corresponding to representative genes up-regulated at the transcript level through RNA-seq (n = 4, a representative blot is shown). Rig-I, TRIM25, DDX1, and RPA2 levels are shown to be increased. (*E*) R-loops are responsible for ~50% of the DNA breaks within CIN 612 cells. COMET assays were performed on HFKs and CIN 612 cells either transduced with a scrambled shRNA sequence, depleted of RNase H1 (shRNH1), or overexpressing RNase H1 (o/eRNH1). Tail moment [Tail length (px) × Tail DNA %] was calculated from three independent experiments. Individual points are plotted with the bars representing a 95% CI from the mean (n = 50; a representative graph is shown).

The effect of increased expression of RNase H1 on viral genome maintenance was next examined by Southern blot analysis (Fig. 4*A*). CIN 612 cells overexpressing RNase H1 substantially reduced viral episomes relative to the scramble control cells after as early as one passage. Furthermore, the levels of viral transcripts for E6, E7, and E1 were reduced by 60 to 80% that of the control CIN 612 cells (Fig. 4*B*). RNA-seq was performed on RNase H1 overexpressing CIN 612 cells to examine how reducing R-loops affected cellular gene expression. The most significant pathway altered was the innate immune signaling pathway where expression was increased as compared to the control CIN 612 cells and included genes such as DDX58, OASL, TRIM25, IL1A, IFIT2, and IFIT3 (Fig. 4*C*). In addition, transcriptional regulators such as p73, EGR1, KLF15, STAT4, and E2F2 were found to be down-regulated. Western blot analysis confirmed the upregulation of DDX58 and TRIM25 at the protein level; both of which are inhibitory to HPV replication (51) (Fig. 4*D*). These data indicate that overexpression of RNase H1 and the resultant reductions in R-loops within HPV-positive cells is important for regulating immune signaling and gene expression.

### DNA Breaks in HPV-Positive Cells Are Caused by R-loop Formation.

A major activity associated with the aberrant formation or resolution of R-loops is the induction of DNA breaks (20). HPVs have been shown to induce high levels of DNA breaks in cells which leads to the activation of DNA repair pathways (20), so we investigated if high levels of R-loops could be a major source. For this analysis, COMET assays were performed using CIN612 cells that were either depleted or overexpressing RNase H1 and compared to HFKs and the scramble control CIN 612 cells. The scramble control CIN 612 cells exhibited about an eightfold greater DNA breaks compared to HFKs, consistent with previous reports (Fig. 4*E*). When RNase H1 was depleted using shRNAs from CIN 612 cells, there was an approximate 2.5-fold further increase in DNA breaks compared to the scramble control. In contrast, overexpression of RNase H1 reduced breaks over 50%. These data indicate that R-loops provide a source for DNA breaks within HPV-positive cells, and that the altered R-loop homeostasis within HPV-positive cells is responsible for over 50% of the DNA breaks.

### HPV 31 E6 Induces R-loop Formation in HFKs.

**Fig. 5.**
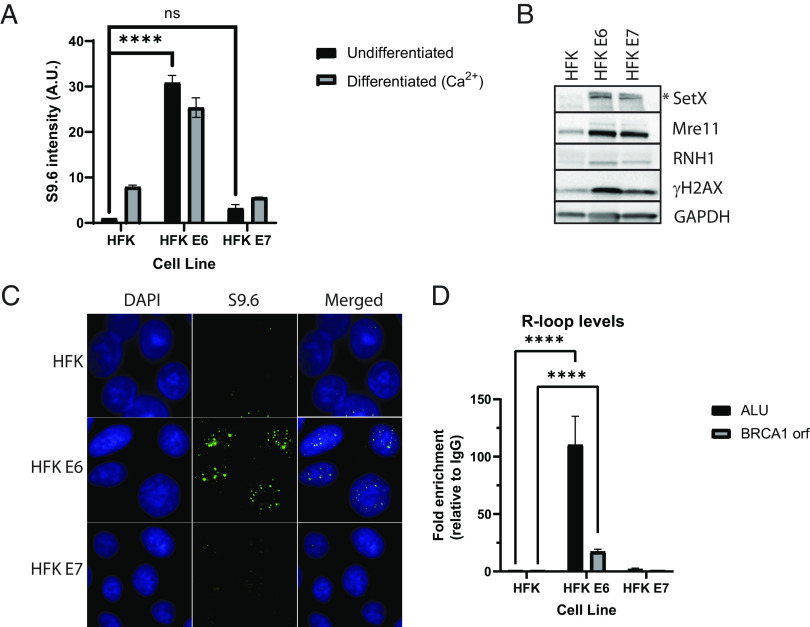
HPV 31 E6 is the primary viral factor responsible for increased levels of R-loops. (*A*) Keratinocytes stably infected with retroviruses expressing HPV 31 E6 or E7 were examined for R-loop levels by S9.6 dot-blot analysis. UD or D HFKs infected with empty vector control, HFK E6, or HFK E7 cells were examined (n = 3; ns, not significant; ****P < 0.0001). (*B*) Western blot analysis for R-loop regulatory factors SetX, Mre11, RNase H1, and gH2AX in UD HFKs, HFK E6, or HFK E7 cells are shown (n = 3; a representative image is shown). (*C*) E6 expression induces nuclear R-loop puncta in HFK E6 expressing cells similar to those seen in HPV-positive cells. Immunofluorescence analysis for S9.6 antibody and DAPI is shown (n = 3; a representative field is shown). (*D*) DRIP analysis of HFKs, HFK E6, or HFK E7 analyzing R-loop levels on ALU respective elements or BRCA1 coding sequences is shown for HFKs, HFK E6, and HFK E7 (n = 3; ****P < 0.0001).

Our studies suggest that the high levels of R-loops in HPV-positive cells may provide important functions in viral life cycle, and it was important to determine whether this increase was a result of viral replication or if the expression of viral proteins alone was sufficient. HFKs expressing E6 or E7 were generated through retroviral transduction and examined for the presence of R-loops by S9.6 dot-blot analysis (Fig. 5*A*). The expression of E6 was found to be sufficient to induce high levels of R-loops in both UD and differentiated (D) states. In contrast, the expression of E7 failed to alter R-loop levels significantly. The levels of R-loops detected in E6-expressing cells were, however, reduced from that seen in cells that stably maintain episomes and could be due to lower levels of E6 expression in these cells or a contribution from other viral proteins. Interestingly the expression of either E6 or E7 resulted in increased levels of the R-Loop regulatory enzymes senataxin, RNase H1, and Mre11 (Fig. 5*B*). The increased levels of these proteins in cells expressing E7, which do not have increased R-loop levels, suggest that this is not the sole determining factor regulating R-loop formation. Immunofluorescence analyses using the S9.6 antibody identified that R-loops formed within nuclear puncta of HFKs expressing E6 cells similar to the staining seen within cells with HPV episomes (Fig. 5*C*), while HFKs expressing E7 more closely resembled the control HFKs. Dot-blot analysis with S9.6 antibodies confirmed that HFKs expressing E6 formed significantly more R-loops than control or E7-expressing HFKs (Fig. 5*D*) and showed increased R-loop formation on BRCA1 as well as ALU sequences. Finally, expression of E6 alone was found to be sufficient to direct recruitment of RNase H1 to the nuclear puncta like that seen in HPV-positive cells, while RNase H1 within E7-expressing HFKs exhibits a pannuclear localization (*SI Appendix*, Fig. S5*A*). The localization of other proteins involved in R-loop resolution like senataxin was however not altered by E6 (*SI Appendix*, Fig. S5*B*). RNase H1 binding to cellular sequences was approximately 3-fold higher in E6 expressing cells than HFKs expressing E7or HFKs (*SI Appendix*, Fig. S5*C*).

**Fig. 6.**
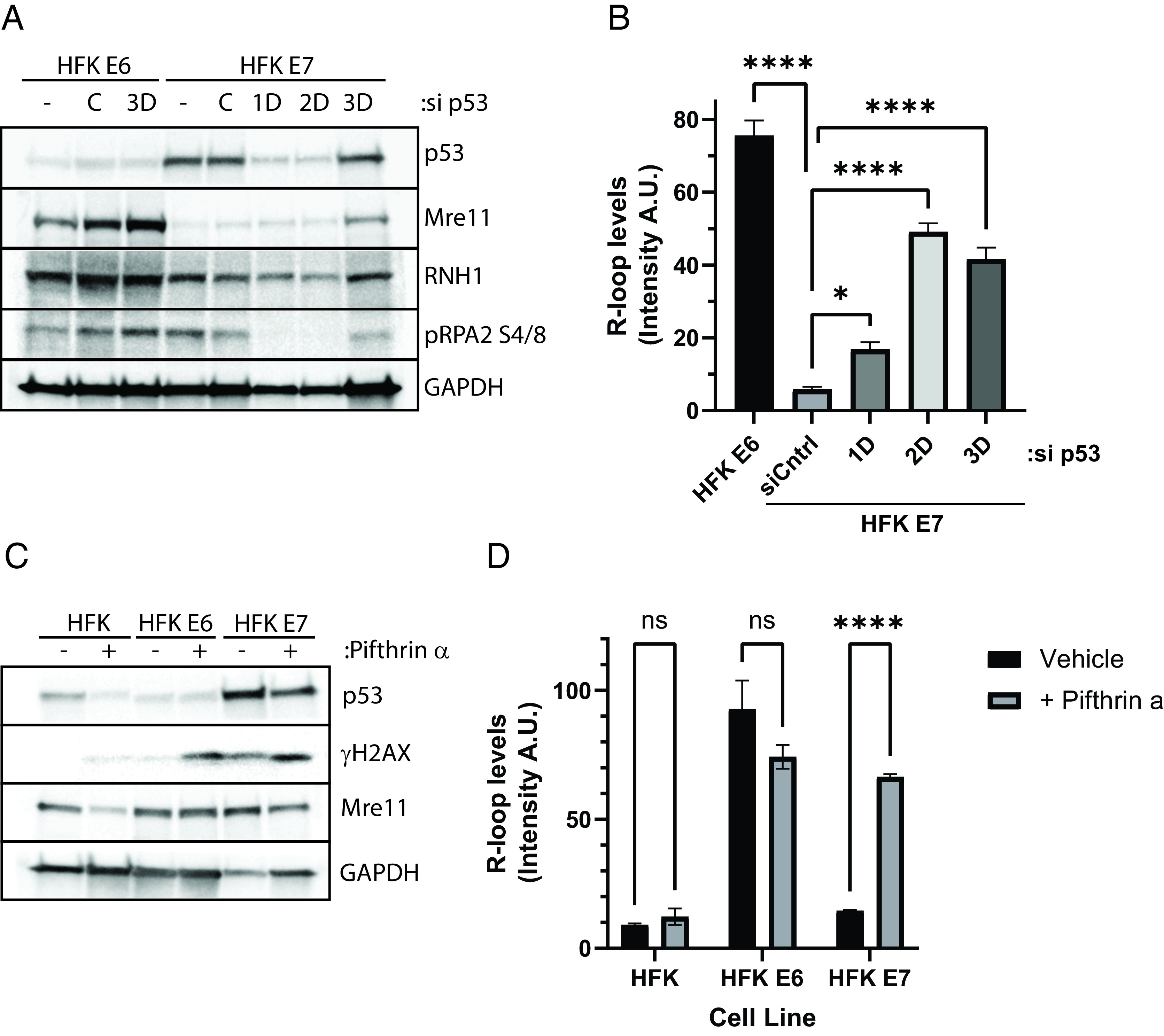
p53 inactivation or depletion drives R-loop formation within HFKs expressing HPV 31 E7. Transient depletion of p53 with transfected siRNAs in HFK E7 cells increases R-loop levels. (*A*) HFKs expressing E6 or E7 either not transfected (−), transfected with an siCntrl vector (C), or transfected with siRNA targeting p53. Samples were collected 24 to 72 h posttransfection and silencing of p53 was validated by western blot analysis. (*B*) S9.6 dot-blot analysis of the same experimental samples as described above (*P < 0.05; ****P < 0.0001). Assays were repeated 4 times with similar results. Inhibition of p53 using pifithrin α similarly increases R-loop levels within E7 expressing HFKs (*C* and *D*). Western blot analysis of HFK, HFK E6, and HFK E7 cells treated with pifithrin a for 24 h (a representative image is shown, n = 3). (*D*) S9.6 dot-blot analysis of the same experimental samples as described above in *C* (n = 3; error bars represent the SEM). Ns, not significant; ****P < 0.0001.

The oncoprotein E6 has many functions, including the capability to degrade and inactivate p53 (52–54). To determine whether the decreased p53 levels seen in E6 expressing HFKs were responsible for R-loop formation, we transiently depleted p53 using siRNA in HFKs expressing E7, which by themselves exhibit high levels of p53. Depletion of p53 steady state levels was observed for 2 d with a restoration of repression on day 3 (Fig. 6*A*). Decreasing p53 levels correlated with decreasing levels of several proteins important for R-loop resolution like Mre11 and RNase H1. R-loop levels were significantly increased within these p53 depleted cells after 1 d, peaked on day 2, and decreased slightly on day 3 (Fig. 6*B*). These data support the hypothesis that inactivating p53 via E6 drives R-loop formation within HPV-positive cells.

Pifithrin-α is an inhibitor of p53’s transcriptional activity and its effect on R-loop formation in E7 cells was examined (55). HFK, HFK E6, or HFK E7 cells were treated with pifithrin-α and R-loop levels were assessed by S9.6 dot blot while p53 levels examined by western blot. Consistent with previous findings, E7-expressing cells exhibited high levels of p53 while E6-expressing cells had low levels (Fig. 6*C*). Treatment of E7 cells with pifithrin-α induced high levels of R-loops. In contrast, inhibition of p53 with pifithrin-α in HFKs did not increase R-loop levels indicating the effect may be specific to E7 expressing cells. These experiments confirm that repression of p53 function is critical for induction of high levels of R-loops in HPV-positive cells.

## Discussion

R-loops provide important physiological functions in regulating transcription as well as other processes; however, improper formation or turnover of these structures leads to conflicts with replication machinery, resulting in genome instability and DNA breaks (1–3). Our studies show that up to 50-fold higher levels of R-loops are present in cells that maintain high-risk HPV episomes as compared to primary keratinocytes. These R-loops are formed not only on viral genomes but also on cellular sequences such as repetitive ALU elements and regulatory elements for genes such as BRCA1 (56, 57). The sites at which R-loops form are similar to those detected in normal cells. In HPV-positive cells, these high levels of R-loops were found to correlate with efficient viral replication and transcription. HPV oncoproteins induce high levels of DNA breaks to activate ATM and ATR damage repair pathways to facilitate viral replication and our studies indicate that over 50% of these breaks are associated with R-loop formation. The increased levels of R-loops are not only seen in cells with HPV episomes but also in squamous cell cervical cancers in vivo. These structures, therefore, provide important functions in the pathogenesis of HPV infections.

The formation and resolution of R-loops is mediated by enzymes such as RNase H1, senataxin, DDX11, and Mre11. The enhanced levels of R-loops present in HPV-positive cells are, however, not the result of reduced levels of these enzymes as they are also increased in these cells. RNase H1 exhibited a pannuclear distribution in HFKs and, while this was also seen in HPV-positive cells, it was also present in puncta as well as at high levels in nucleoli. Interestingly, HPV-positive cells also exhibited increased numbers of nucleoli that contained RNase H1, which may reflect a role of RNase H1 in cooperating with Pol1 in directing ribosomal RNA transcription (42). Furthermore, R-loops were detected bound to HPV genomes at the early promoter (p97) and polyA site but not to E7 or E2 coding sequences. RNase H1 was also present at sites with enriched R-loops levels (ALU sequences and the URR), implicating RNase H1 as actively regulating viral and cellular R-loops within these cells.

A reduction in p53 levels was found to be responsible for inducing high levels of R-loops. While E6 and E7 can cause DNA breaks (20), only E6 was able to induce high levels of R-loops. A primary function of E6 is the impairment of p53 function and our studies demonstrate that knockdown of p53 in cells expressing only E7 leads to induction of high levels of R-loops, implicating it as a key regulator. p53 has been reported to regulate the levels of methyl donor S-adenosylmethione (SAM) which in turn controls histone H3 lysine methylation at repetitive satellite DNAs (58). In p53 deficient pancreatic cancer cells, SAM is repressed resulting in R-loop formation at these repetitive sites, but whether this is a primary mode of action in HPV-positive cells is unclear. While E6 expressed from a retroviral promoter was sufficient to induce R-loop formation, the levels were reduced from that seen in cells that maintain complete viral episomes, which may indicate lower expression of E6 from integrated transgenes compared to episomes or that another viral factor also contributes.

The enhanced levels of RNase H1 which resulted in high R-loops were found to be critical for HPV replication and transcription. RNase H1 removes the RNA moiety from the RNA:DNA hybrid of an R-loop and knockdown with shRNAs leads to increased R-loop levels. It is not possible to directly alter R-loop levels, and this can only be achieved by modulating the amounts of its regulatory enzymes such as RNase H1. Changing the level of RNase H1 has been characterized in numerous studies as the gold standard method to modulate R-loops (42, 59, 60). In HPV-positive cells, depletion of RNase H1 increased total R-loop levels by ~8-fold and this resulted in greater amounts of DNA breaks along with impaired viral replication and gene expression. R-loops have been shown to regulate chromatin organization by modulating histone methylation patterns which in turn modulates transcription (61, 62). In our studies, reducing RNase H1 led to enhanced levels of R-loops and decreased expression of cellular genes, particularly those in DNA damage repair pathways. This included FANCD2, which has been shown to be necessary for viral replication (26). FANCD2 binds to HPV promoter sequences and has also been shown to form complexes with R-loops suggesting that its association with viral genomes may be mediated through these structures (26). Another factor whose expression was reduced by the high levels of R-loops was the DNA repair kinase, ATR, whose activation has been shown to be necessary for viral replication (27). Despite the presence of higher levels of DNA breaks in RNase H1 knockdown cells, activation of repair pathways was not seen due to repressed transcription of critical DNA damage repair genes. Finally viral gene expression, including that of E1, was also reduced by the reductions in RNase H1 and high amounts of R-loops, further contributing to impaired viral replication. These experiments identify multiple factors regulated by RNase H1 and R-loops that are critical for viral gene expression and replication.

While knockdown of RNase H1 leads to increased amounts of R-loops, overexpression reduced levels only slightly higher than those observed in normal HFKs. This reduction in R-loops from RNase H1 overexpression correlated with decreased viral transcription and episomal copy numbers along with increased expression of cellular genes responsible for innate immune signaling. In addition, the levels of DNA breaks were decreased by ~50%, which resulted in reduced activation of DNA repair pathways. Previous studies have shown that a substantial number of breaks result from the action of topoisomerases such as TOP2 β and our studies indicate that a substantial part of the remainder are due to R-loops (63). The impairment observed in viral gene expression correlated with a reduction in R-loops and suggests that forming these structures on HPV episomes is important for viral transcription. Alternatively, it is possible that increased expression of cellular immune response genes like Rig I and TRIM25 act to hinder viral transcription (51). Overall, these studies indicate that R-loop homeostasis in HPV-positive cells is critical for regulating the viral life cycle, as reducing or increasing R-loop levels impairs viral transcription and replication, directly or indirectly, by altering the expression of critical cellular genes.

## Materials and Methods

### Isolation of HFK and Cell Culture.

HFK were isolated from deidentified neonatal foreskins provided by the Skin Disease and Research Core at Northwestern University as previously described (20). Cells were cultured as previously described (20, 64, 65). Briefly, HFKs and CIN 612 cells which were isolated from deidentified biosamples and stably maintain HPV 31 episomes were cocultured in E-media with NIH-3T3-J2 fibroblasts (J2s), which were growth arrested with mitomycin C. J2s and HEK-293T cells were cultured in Dulbecco’s modified Eagle’s medium with 10% Fetal bovine serum and 1% pen-strep. HFKs stably maintaining HPV 31 episomes were generated as previously described (66). HFKs stably expressing viral oncogenes E6 or E7 were generated as previously described (20). Cells were treated with Pifithrin-α (100 mM) for 24 h to assess the effect of p53 inhibition on R-loop levels.

### Generation of Cell Lines that Stably Express shRNAs or RNase H1-eGFP.

Plasmids encoding shRNA sequences targeting RNase H1 were purchased from Sigma. The sequences of the RNAs targeted RNase H1 are listed in *SI Appendix*, Table S3. Lentiviruses were generated with each of the four shRNA encoding plasmids in HEK-293T cells using the 2nd generation AddGene system. CIN612 cells were transduced with the various lentiviruses and selected using puromycin (2 mg/mL). Depletion of RNase H1 was validated by western blot analysis and fold change was quantified via densitometry using ImageJ (NIH). Overexpression of RNase H1 was achieved by using the pEGFP-RNase H1 vector [a gift from Andrew Jackson and Martin Reijins (Addgene plasmid #108699)]. Lentiviruses were generated using this vector or empty vector control using the 2nd generation AddGene system in HEK-293T cells. CIN 612 cells were then transduced and assessed for RNase H1 expression by immunofluorescence and western blot analysis of GFP.

### Calcium-Induced Differentiation.

5 × 10^7^ cells were collected and plated into 10-cm dishes containing M154 media containing 0.07 mM CaCl_2_ supplemented with human keratinocyte growth serum (HKGS) (LifeTech). After 24 h, the media were changed to that containing 0.03 mM CaCl_2_. On the third day, M154 medium without HKGS and containing 1.5 mM CaCl_2_ was added to confluent monolayers of keratinocytes. Differentiating keratinocytes were incubated for up to 72 h at 37 °C before being harvested for downstream analyses. Validation of differentiation was assessed through a comparison of K10 levels between UD and differentiated cell lysates.

### siRNA Transfections.

Transient silencing of p53 expression was performed in HFKs expressing either HPV31-E6 or -E7 with transfected siRNAs according to protocols from Santa Cruz Biotechnology. Cell lysates were collected 24 to 96 h posttransfection and assessed by western blot analysis for p53 steady-state levels and dot-blot analysis for R-loops.

### S9.6 Dot-Blot Analysis.

DNA was purified from cell lysates using PhenolChloroform extractions and spotted onto a positively charged membrane (Zeta-probe). Membranes were then blocked with 5% Bovine Serum Albumin (BSA) in TBST (Tris-buffered saline Tween 20) before being probed with the S9.6 anti-RNA:DNA hybrid antibody (Millipore) overnight at 4 °C. The following day, membranes were washed with TBST, probed with secondary antibody for 1 h at RT, and developed using enhanced chemiluminescence (ECL) (Fisher, 4500085). Images were taken using an Odyssey Fc LiCor (LiCor BioSciences).

### Western and Southern Blot Analyses.

Western blot analysis was performed as previously described (64) using the antibodies listed in *SI Appendix*, Table S1. Southern blot analysis was performed as previously described (64).

### Immunofluorescence Analysis.

2.25 × 10^5^ cells were plated onto a 4-chamber slide (MatTek). The following day, cells were either fixed with 4% paraformaldehyde or methanol and stored in phosphate buffered saline (PBS) overnight at 4 °C. Chambers were permeabilized in 0.5% TritonX-100 and blocked with 3% BSA in PBS. Samples were probed with antibodies listed in *SI Appendix*, Fig. S1 before staining with DAPI and secondary antibodies. After mounting with mounting medium (VectaShield), chambers were imaged with a Ti2 eclipse (Nikon).

### Immunofluorescence Staining of Paraffin Sections.

Six cross-sections of the same tissue from high-grade cervical carcinomas (n = 3) were formalin-fixed and paraffin embedded. Immunohistochemistry (IHC) was also performed to identify the margins between normal tissue and tumor. Immunofluorescence of paraffin-embedded sections was performed as previously described (67). Heat antigen retrieval was performed at 60 °C overnight. Specificity of S9.6 staining was analyzed by digesting dewaxed, permeabilized tissues with RNase T and III (2.5U, Invitrogen and ThermoFisher) or RNase H (2.5U, ThermoFisher) for 1 h.

### RT-qPCR Analysis.

RNA was extracted using the Qiagen RNeasy Kit from confluent 10 cm dishes. Reverse transcription reactions were then performed on 20 ng of RNA using the iScript cDNA Synthesis Kit (BioRad). Real-time PCR was performed using a LightCycler 480 system (Roche) with primer sets mapping to the E1, E6, and E7 open reading frame (*SI Appendix*, Table S2).

### Neutral Comet Assay.

COMET assays were performed following the manufacturer’s instructions (Trevigen, cat. No. 4250-050-K). Briefly, ~50,000 cells were combined with low-melt agarose and spread across the CometSlide. Once dry, cells were lysed for 1 h at 4 °C. Cells were equilibrated to 1x neutral electrophoresis buffer, and DNA was resolved on an electrophoresis slide tray for 45 min at 21 V, 4 °C. DNA was precipitated and stained with SYBR Gold for 30 min before imaging on a Ti2 Eclipse microscope (Nikon). Tail moments were calculated using the open-source software, CometScore 2.0 (% DNA in tail × tail length = tail moment).

### Chromatin Immunoprecipitation Assays.

Cells from a confluent 10-cm dish were cross-linked with 1% formaldehyde and collected in RIPA buffer. Samples were analyzed as previously described (20). Primers used for qPCR analysis are listed (*SI Appendix*, Table S2).

### DRIP Assays.

1 × 10^7^ cells were harvested and collected in Southern lysis buffer before being treated with RNase A (5 ng/mL) and Proteinase K (7.5 ng/mL) at 37 °C overnight. DNA was purified from these samples using phenol–chloroform extractions and 25 to 50 mg of DNA was used for each sample. DNA was sheared using a Bioruptor (Diagenode) on high power, 30 s on/90 s off cycles for 20 min or digested using 1U of mung bean nuclease for 1 h at 37 °C. Input DNA was removed before loading the samples into preblocked magnetic beads in IP buffer containing 2 mg of the RNA:DNA hybrid antibody. Immunoprecipitations were allowed to incubate overnight at 4 °C while rotating. The next day, samples were washed 8 times with RIPA buffer for 5 min while rotating. One wash in TE buffer was performed before samples were eluted for 10 min at 65 °C in 10% sodium dodecyl sulfate (SDS), 10 mM Tris pH 7.4, 50 mM ethylenediaminetetraacetic acid (EDTA). DNA was purified from these elutions using a PCR purification kit (Qiagen) and stored at −20 °C. Samples were then analyzed by qPCR (primers used listed in *SI Appendix*, Table S2).

### RNA-seq Analysis.

1 × 10^7^ keratinocytes were harvested and analyzed by Admera Biosciences (NJ), who performed the RNA extraction as well as sequencing. Following RNA extraction, mRNA was sequenced using the Illumina platform. Data analysis was also performed by Admera Biosciences (NJ). DeSeq2 reads of genes differentially expressed between control CIN 612 cells and CIN 612 cells depleted of or overexpressing RNase H1 are provided (*SI Appendix*, Tables S3 and S4). Biostatistical analyses were performed by Admera Biosciences (NJ) services. RNA-seq data were deposited to the GEO database (NCBI).

### Preparation of Digital Figures and Statistical Analyses.

Statistical analysis was performed using student *t* tests and multiple-way ANOVAs on GraphPad Prism9 software (CA, USA). Graph preparation was performed using GraphPad Prism9 software. Final images were assembled using Adobe Photoshop and Illustrator (CA, USA).

## Supplementary Material

Appendix 01 (PDF)

## Data Availability

Fastq files and metadata have been deposited in the National Center for Biotechnology Information (NCBI) Gene Expression Omnibus (GEO) (46).
