# Supplementary material for: p53-dependent R-loop formation and HPV pathogenesis
Source: Proc Natl Acad Sci U S A. 2023 Aug 23;120(35):e2305907120. doi: 10.1073/pnas.2305907120 (PMC10467572; doi:10.1073/pnas.2305907120)
Supplement: Supplementary file 1 — Appendix 01 (PDF) [file pnas.2305907120.sapp.pdf]

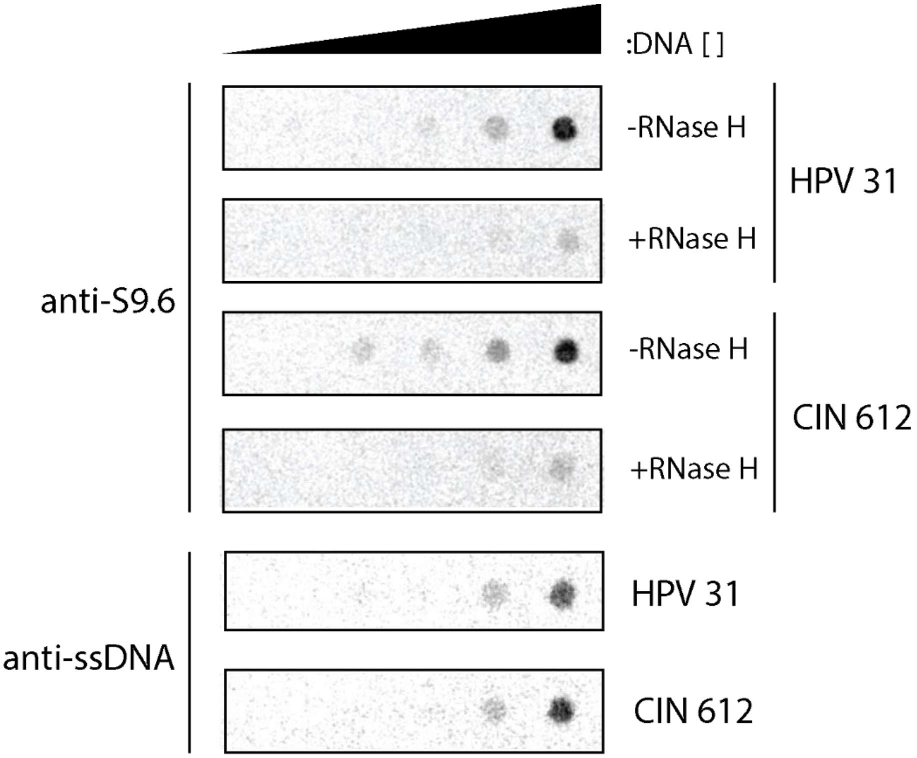

Supplemental Figure 1 – S9.6 specificity for R-loop detection through dot blot analysis. The S9.6 antibody is specific for R-loops in HPV positive cells. Nucleic acids were extracted from HFK 31 and CIN 612 cells and either left untreated or treated with RNase H prior to S9.6 antibody dot blot analysis. Increasing DNA concentrations were loaded, ranging from 25 to 800ng. A subset was denatured and probed with a ssDNA antibody to control for DNA loading, while the rest were probed with the S9.6 antibody. A representative image of three biological replicates is shown.

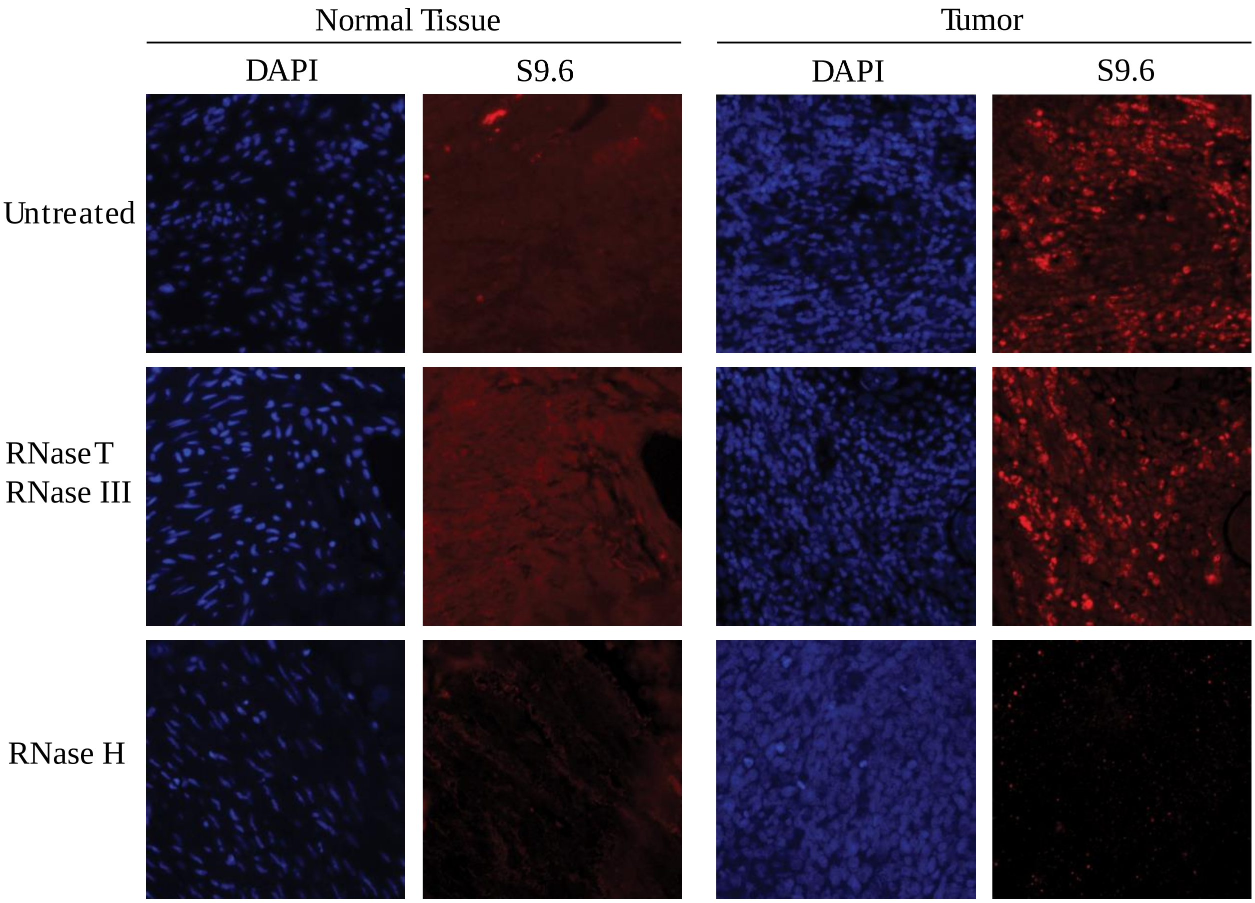

Supplemental Figure 2 – S9.6 staining of fixed tissues is sensitive to RNase H treatment. Immunofluorescence analysis was performed on paraffin-embedded tissue from high-grade cervical carcinomas, including normal tissue at adjacent margins. Immunohistochemistry identified normal tissue and tumor. Tissues were permeabilized and either left untreated (top), digested with 2.5U of RNase T and III (middle), or digested with 2.5U RNase H (bottom). Cross sections of the same tissue were used for immunofluorescence analysis with antibodies recognizing R-loops (S9.6) and DAPI.

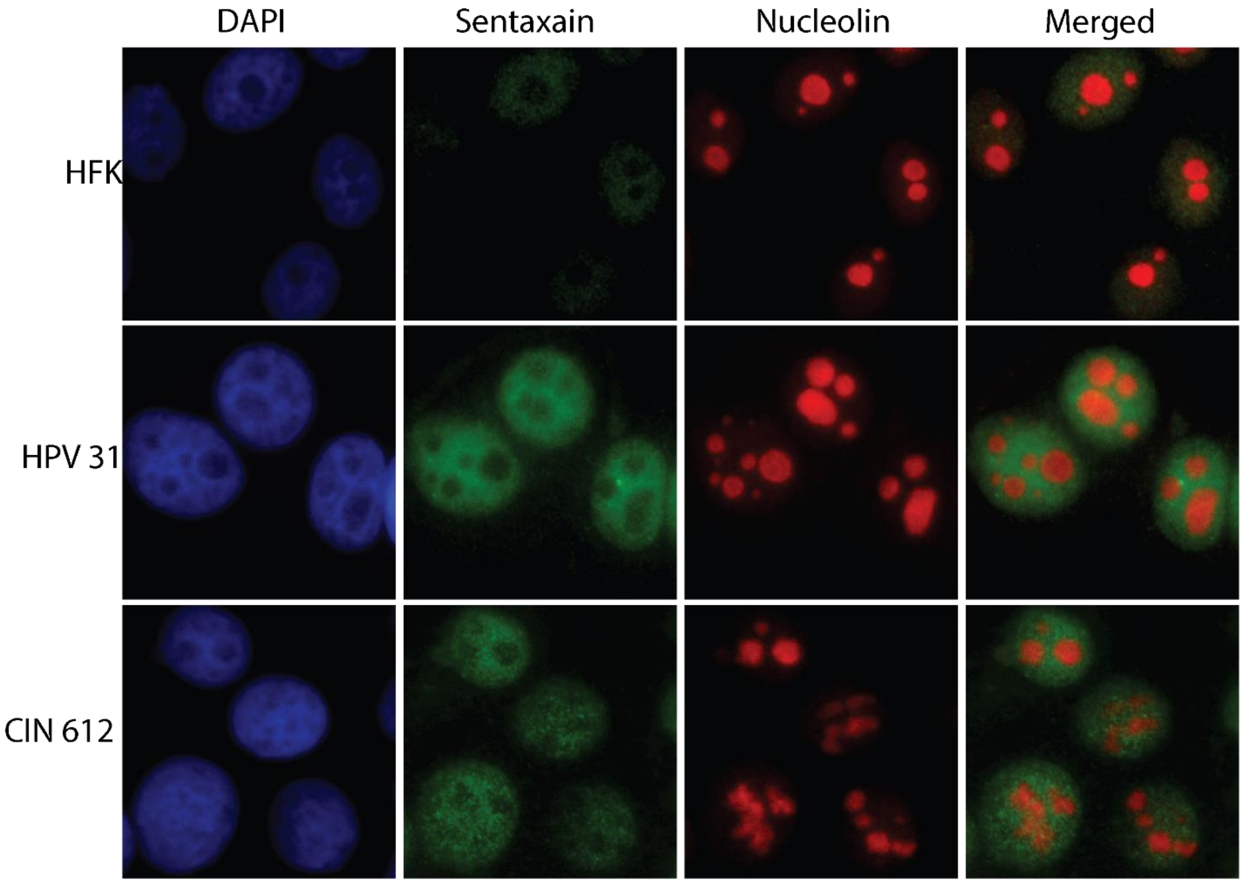

Supplemental Figure 3 – Senataxin is not recruited to nucleoli within HPV positive cells. Immunofluorescence analysis of undifferentiated HFKs, HFK 31, and CIN 612 cells using the senataxin and nucleolin antibody (n=3, a representative field is shown).

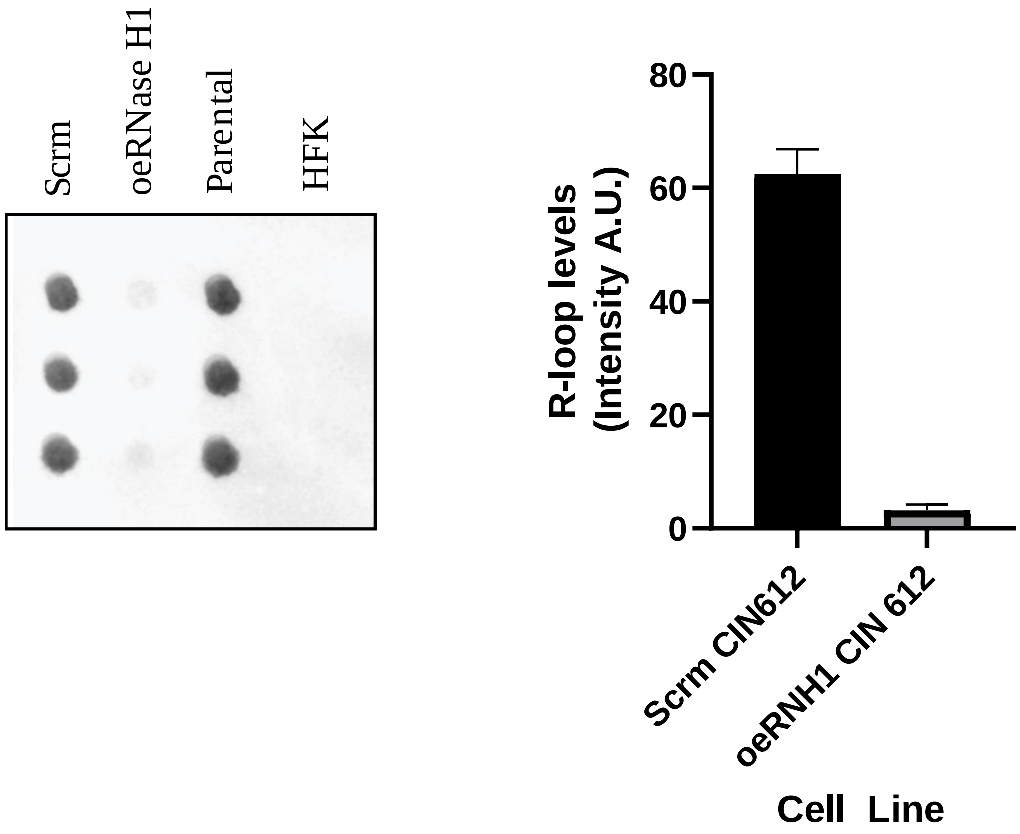

Supplemental Figure 4 – Overexpression of RNase H1 reduces R-loop levels within CIN 612 cells. Overexpression of RNase H1 substantially reduces R-loop levels within HPV positive cells. Dot blot analysis of Scrm, o/eRNH1, and parental CIN 612 cells to HFKs using the S9.6 antibody (n=4; a representative image is shown). The data is plotted as the average of four replicates with the error bars representing SEM.

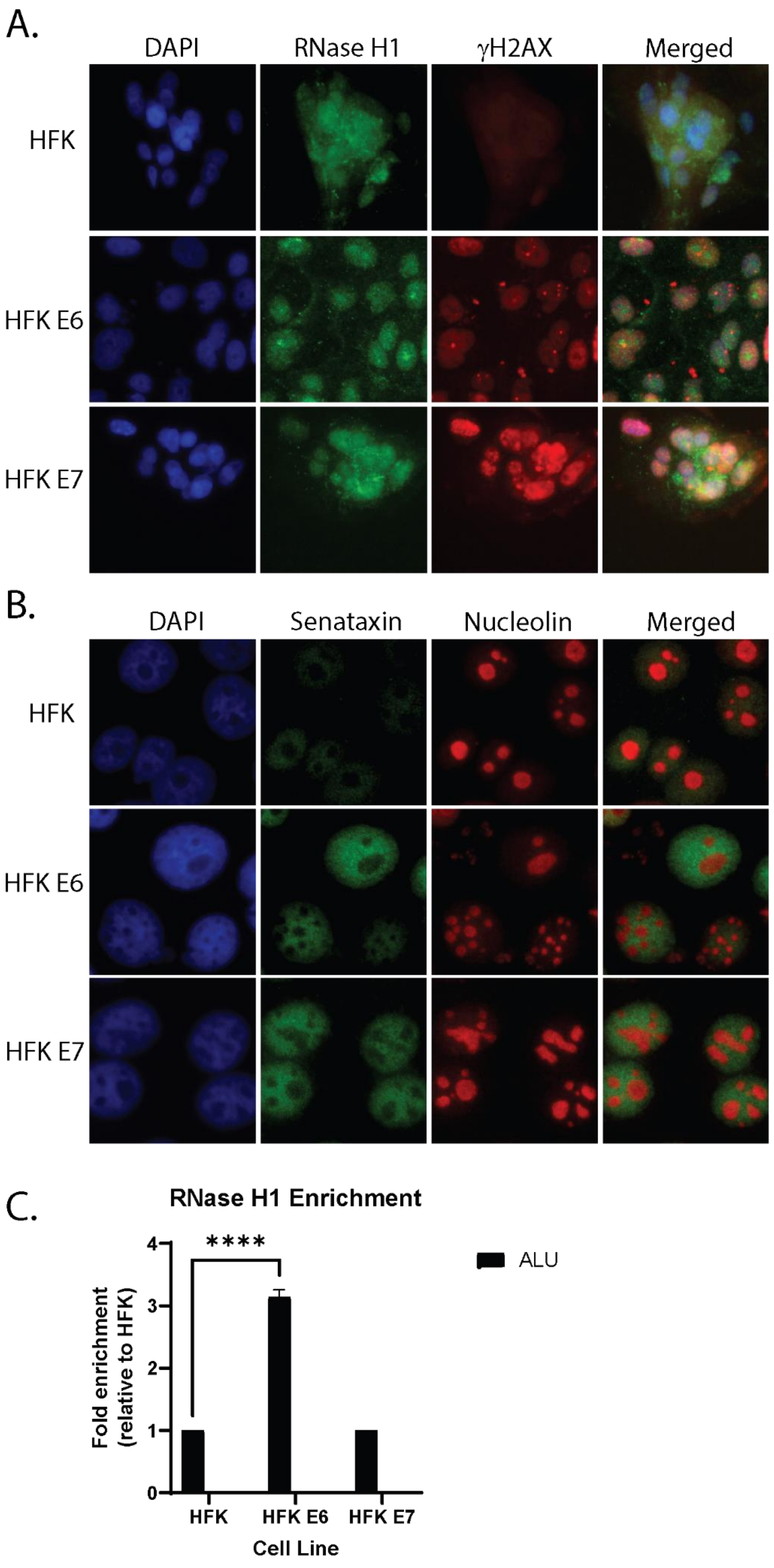
A.
DAPI
RNase H1
γH2AX
Merged
HFK
HFK E6
HFK E7
B.
DAPI
Senataxin
Nucleolin
Merged
HFK
HFK E6
HFK E7
C.
RNase H1 Enrichment
****
ALU
Fold enrichment (relative to HFK)
0
1
2
3
4
HFK
HFK E6
HFK E7
Cell Line

34

Supplemental Figure 5 – HPV 31 E6 alters RNase H1 subcellular localization. RNase H1 but not senataxin exhibits an altered subcellular localization in HFK E6 cells (A) Immunofluorescence analysis of RNase H1 and γH2AX or RNase H1 and nucleolin in undifferentiated HFK, HFK E6, and HFK E7 cells (n=3; a representative field is shown). (B) Immunofluorescence analysis of undifferentiated HFKs, HFK E6, and HFK E7 cells using antibodies to senataxin and nucleolin antibody (n=3, a representative field is shown). (C) Chromatin immunoprecipitations of sequences bound by RNase H1 in HFKs, HFK E6, and HFK E7. Fold enrichment was calculated as (RNase $H1_{x}$/IgG_{x})/(RNase $H1_{HFK}$/IgG_{HFK}) where x is the Ct value from the HFK E6 or HFK E7 cells. The data is plotted as the average of three biological replicates, and the error bars represent the SEM (****, p<0.0001).

Supplemental Table 1 – Antibodies used within this study.

Antibody	Host Animal	Dilution for immunofluorescence	Dilution for Western blot	Distributor, cat. No.
**RNase H1**	Rabbit, polyclonal	1:200	1:1000	ThermoFisher, 15606-1-AP
**RNase H1**	Mouse, monoclonal	N/A	1:100	Santa Cruz Biotechnology, sc-365267
**ssDNA**	Mouse, monoclonal	N/A	1:1000	Millipore Sigma, MAB3868
**DNA-RNA Hybrid (S9.6)**	Mouse, monoclonal	1:200	1:1000	Millipore Sigma, MABE1095
**Senataxin**	Rabbit, polyclonal	1:200	1:1000	Novus Biologicals, NBP194712
**GAPDH**	Mouse, monoclonal	N/A	1:4000	Santa Cruz Biotechnology, sc47724
**Mre11**	Rabbit, polyclonal	N/A	1:1000	Cell Signaling Technologies, 4896S
**DDX11**	Mouse, monclonal	1:400	1:1000	Santa Cruz Biotechnologies, sc271711
**Mouse IgG**	Mouse, monoclonal	N/A	N/A	Fisher Scientific, C1540000115
**GFP (B-2)**	Mouse, monoclonal	1:50	1:200	Santa Cruz Biotechnology, sc9996
**pATR (ser1981)**	Rabbit, polyclonal	N/A	1:500	Cell Signaling Technologies, 13050S
**ATR**	Mouse, monoclonal	N/A	1:100	Santa Cruz Biotechnologies, sc515173

**Nucleolin**	Mouse, monoclonal	1:400	N/A	Invitrogen, 39-6400
**γH2AX (ser139)**	Rabbit, monoclonal	N/A	1:1000	Cell Signaling Technologies, 9718S
**γH2AX**	Mouse, monoclonal	1:200	N/A	Fisher, 05-636-IMI
**pRPA32 (ser8)**	Rabbit, polyclonal	N/A	1:500	Cell Signaling Technologies, 83745S
**RPA32**	Rabbit, monoclonal	N/A	1:1000	Abcam, ab2175
**FancD2**	Rabbit, monoclonal	N/A	1:500	Abcam, 178705
**TRIM25**	Rabbit, polyclonal	N/A	1:1000	Abcam, ab167154
**Rig-I**	Rabbit, monoclonal	N/A	1:1000	Cell Signaling Technologies, 3743S
**Anti-Rabbit IgG, HRP-linked**		N/A	1:3000 – 1:5000	Cell Signaling Technologies, 7074
**Anti-Mouse IgG, HRP-linked**		N/A	1:3000 – 1:5000	Cell Signaling Technologies, 7076
**Goat anti-Mouse IgG (H+L) Alexa Fluor™ 594**		1:400	N/A	ThermoFisher, A-11032
**Goat anti-Rabbit IgG (H+L) Cross-Adsorbed Secondary Antibody, Alexa Fluor™ 488**		1:400	N/A	Invitrogen, A-11008
**Goat anti-Mouse IgG (H+L), Superclonal™ Recombinant Secondary Antibody, Alexa Fluor™ 488**		1:400	N/A	Invitrogen, A28175
Goat anti-Rabbit IgG (H+L) Highly Cross-Adsorbed Secondary Antibody, Alexa Fluor™ Plus 594		1:400	N/A	Invitrogen, A32740

Supplemental Table 2 – Primer sets used within this study.

Primer name	Sequence (5' to 3')
**ALU Forward**	ACG AGG TCA GGA GAT CGA GA
**ALU Reverse**	CTC AGC CTC CCA AGT AGC TG
**URR Forward**	GAT GCA GTA GTT CTG CGG TTT
**URR Reverse**	TAT GTT GGC AAG GTG TGT TAG G
**E6 Forward**	GAC CTC GGA AAT TGC
**E6 Reverse**	AAC ATG CTA TGC AAC GTC CTG
**E7 Forward**	AAT TAC CCG ACA GCT CAG ATG
**E7 Reverse**	GGC ACA CGA TTC CAA ATG AG
**E2 Forward**	TAC TGT TGT GGA AGG GCA AG
**E2 Reverse**	TCC CAG CAA AGG ATA TTT CGT C
**E1 Forward**	GAC AGA CAG ACA GGG G
**E1 Reverse**	CCC GCT GTC TGG AAG TTC
**Late PolyA Forward**	GCG TGT GTA CTT GTA
**Late PolyA Reverse**	GCA ACC GAA AAC GGT TAG G
**P97 Forward**	GGG AGT GAC CGA AAG TGG
**P97 Reverse**	CGT GTG GTG TGT CGT CC
**Early PolyA Forward**	GGT ATT GGT ATT GGT ATT GG
**Early PolyA Reverse**	ACC CAT ACT ACC ATA CCT TA
**BRCA1 Forward**	GGC TTC TAA CAG CTA CCC TTC
**BRCA1 Reverse**	CTT CTG GAT TCT GGC TTA TAG GG

Supplemental Table 3 – shRNA sequences targeting RNase H1

Code (TRC Number)	Sequence
**shRNH1 3-1 (N0000049783)**	5' – GCCGTATGCAAAGCACATGAA – 3'
**shRNH1 5-1 (N0000049785)**	5' – CCTGGTCATTCGGGATTTATA – 3'
**shRNH1 8-1 (N0000119548)**	5' – CACTCAGGATTTGTGGGCAAT – 3'
**shRNH1 3-2 (N0000291902)**	5' – GCAAAGCCATTGAACAAGCAA – 3'
